# A rare case of centre sparing corneal opacity with unilateral Reis-Buckler dystrophy

**DOI:** 10.11604/pamj.2023.45.91.40483

**Published:** 2023-06-21

**Authors:** Ankit Gupta, Sachin Daigavane

**Affiliations:** 1 Department of Ophthalmology, Jawaharlal Nehru Medical College and Acharya Vinoba Bhave Rural Hospital, Datta Meghe Institute of Higher Education and Research, Sawangi (M), Wardha, India

**Keywords:** Corneal opacity, opacity, cornea, keratoplasty, ophthalmology

## Image in medicine

One of the main factors affecting eyesight is corneal opacity. There are 1.5 to 2.0 million occurrences of monocular blindness annually, which is attributed to corneal opacity brought on by trauma or corneal ulcers. Trauma, chemical burns, infections, surgeries, and secondary corneal illnesses or disorders, such as corneal dystrophies, can all cause corneal opacity. The primary criteria of whether corneal opacity might resolve spontaneously and the likely time course of resolution are the pathophysiologic mechanisms leading to the opacity following damage. This is a case of a 79-year-old female with complaints of a diminution of vision in both eyes for the past two years. The vision was 6/24P in the right eye and 6/60P in the left eye. Both eyes were pseudophakic. On slit lamp examination, both eyes have peripheral corneal opacity with arcus senilis. The patient had a history of cataract surgery in the right eye 4 years back and in the left eye 3 months back. Upon auto-refraction, the right eye had -1.12S -1.37S x 95 axis, and the left eye had no target due to corneal dystrophy. The fundus examination was within normal limits with no abnormality. The chosen course of treatment for penetrating keratoplasty is a conventional full-thickness corneal transplant technique, in which an opaque cornea is cut using a trephine (a circular cutting tool), and a correspondingly sized section of the donor cornea is taken with a second trephine. The corneal button refers to the portion of the donor cornea that has been removed. The patient's eye is subsequently stitched to the donor tissue. Penetrating keratoplasty can be used to treat dense corneal opacity, which covers the whole corneal layer.

**Figure 1:**
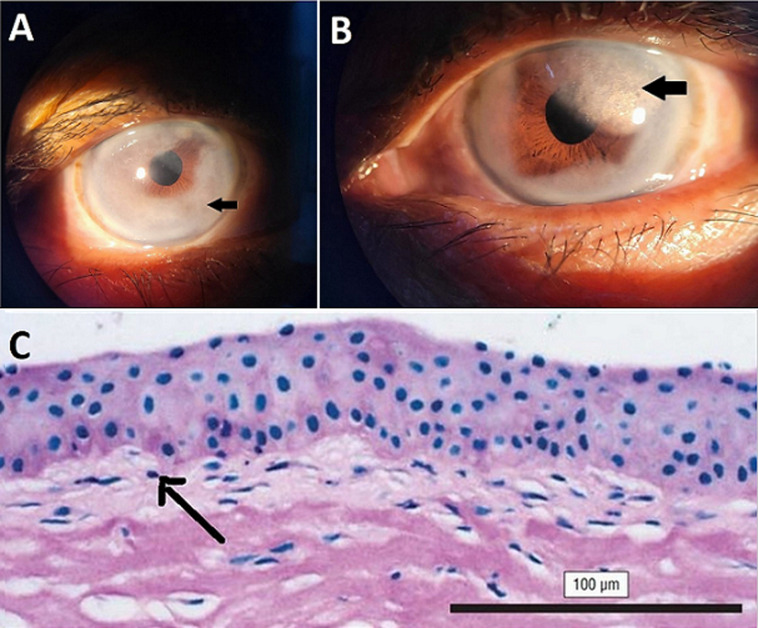
slit lamp picture showing: A) peripheral and paracentral corneal opacity in right eye; B) peripheral and paracentral corneal opacity with Reis-Bucklers corneal dystrophy; C) periodic acid-Schiff (PAS) staining of a corneal specimen from a diagnostic biopsy reveals the absence of the Bowman layer and a band of abnormal connective tissue (marked by arrow) between the corneal epithelium and stroma

